# Intergenerational transmission of family adversity: Examining constellations of risk factors

**DOI:** 10.1371/journal.pone.0214801

**Published:** 2019-04-24

**Authors:** Ingrid Schoon, Gabriella Melis

**Affiliations:** University College London, Institute of Education, London, United Kingdom; University of Wyoming College of Health Sciences, UNITED STATES

## Abstract

This study adopts a socio-ecological approach to examine multiple factors and processes assumed to shape the intergenerational transmission of social disadvantage, including influences of social change, social causation and social selection. Moving beyond approaches focusing on cumulative risk indices, this study uses latent class analysis to examine how different socio-economic and psycho-social risk factors combine within families and to what extent and how constellations of risk are transmitted from one generation to the next. We draw on data collected for the longitudinal and national representative 1970 British Cohort Study, comprising information on more than 11,000 cohort members and their parents. We identified four distinct risk configurations among the parent generation (G1): low-risk families (57.6%), high-risk families (16.3%), high-risk single-parents (24%) and ethnic minority families (2.1%). Within their offspring (G2) we identified five distinct risk configurations: low-risk families (62%), low-risk no-children (15.1%), moderate-risk single parents (10.1%), moderate-risk large families (8.9%), high socio-economic and high psycho-social risk (4%). There is evidence of structural mobility, and the findings suggest that intergenerational transmission of disadvantage is not just a systemic tendency towards social reproduction, but also reflects processes of social change and social selection. We conclude that a socio-ecological model provides a useful framework for a more comprehensive understanding of the multiple processes involved in the transmission of inter-cohort inequality.

## Introduction

Previous research has shown that indicators of parents’ socio-economic and psycho-social risks are significantly and independently associated with their children’s outcomes as adults, although not all family characteristics are equally decisive [1, 2]. By focusing only on single indicators of family adversity, such as parental occupational or educational status, we potentially miss substantial components of family adversity. Different risk factors accumulate and it is usually not one but multiple factors that matter [3–6]. The accumulated adversities in the family of origin have intergenerational consequences across multiple outcomes in their offspring [1, 2]. However, there is a dearth of studies taking into account a range of possible and potentially co-occurring adversities, assessing to what extent these disadvantages cluster across generations.

This study is informed by a socio-ecological model of human development [7], examining a) constellations of risk; and b) the intergenerational transmission of risks. In our analysis we adopt a person- or rather family-centred approach putting forward the hypothesis that there are subgroups of families that share a homogeneous pattern of risk factors. Moreover, we ask if these patterns are transmitted across generations, and how? Previous research suggests that socio-economic and psycho-social problems co-occur [4, 8, 9] and that a person-centred approach can provide a more nuanced understanding of how different risk factors interact. While constellations of early family risk could be established in a couple of US based studies, these studies focused on the association between family risk and outcomes in early childhood or adolescence [10]. Moreover, previous studies have focused on outcomes within a specific domain, such as educational attainment or behavioural adjustment. The contribution of this paper is 1) to examine constellations of adversity experienced across domains in the family of origin (G1) and among their offspring (G2); 2) linking G1 risk exposure to G2 risk exposure (spanning four decades); and 3) examining different mechanisms in the transmission of disadvantage.

We take a longitudinal approach, drawing on data from a nationally representative British Cohort Study born in 1970 (BCS70). We link information collected from parents (G1) of the cohort members during early childhood to information collected from the cohort members themselves (G2) at age 42. When assessing constellations of risk in G1 we focus on risks occurring before school entry of the cohort members (G2), recognizing the importance of early experiences in shaping long-term developmental outcomes [11,12]. Regarding outcomes, we focus on constellations of risks in the second generation, assessing developmental outcomes by age 42, when most cohort members will have completed their education, established themselves in the labour market and have started their own families. Regarding processes of the intergenerational transmission of risk, we test assumptions formulated within theories of social change, cumulative (dis)advantage, and social selection.

## Constellations of family risk

There is consistent evidence to demonstrate the detrimental and long-term effects of exposure to family adversity on the academic and occupational attainment as well as health and wellbeing in the second generation [11–14]. According to theories of cumulative (dis)advantage [15–17] the experience of adversity in the family of origin (G1) increases the risk of experiencing similar adversities in the second generation (G2). The notion of cumulative (dis)advantage is considered as a general “systemic tendency” [17] for inequality across any temporal process (e.g., life course, family generations) in which a favourable relative position becomes a resource that produces further relative gains. The central idea is that inequality is magnified over a life course because individuals or families accumulate different amounts of advantages and disadvantages over time, i.e. those who are initially advantaged are more likely to acquire a good education, leading to a good job, better health, etc., whereby the "advantage" of one individual or group over another grows (i.e., accumulates).

Risks tend to cumulate not only over time–but also across domains. Socio-economic risks, such as low parental education, low occupational status, or unemployment do not appear in isolation, but co-occur with a wider range of other family hardships, such as young motherhood, single parent families, large family size, low quality housing, overcrowding, poor mental and physical health [4, 10]. Moreover, risks are not equally distributed across racial and ethnic groups, with family risk factors being more prevalent among ethnic minority groups [9, 18], emphasising the importance of considering ethnicity as a risk indicator. Although associations between socio-economic family risks and psycho-social functioning of the parents are well documented, there is still a lack of understanding regarding potential patterns and configurations of risk factors that occur within families [19]—and to what extent such configurations of risks are transmitted from one generation to the next.

### Cumulative risk indices

Previous research has recognised the empirical challenges of trying to comprehensively assess the ways in which risk factors co-occur. One approach is the use of cumulative risk indices, which quantifies the number of risks present in a child’s life and establishing associations to a range of outcomes, such as educational attainment, health and wellbeing [3–5]. This approach is based on the assumption that it is the accumulation rather than the content of the risk factor that matters most in shaping children’s development. Cumulative risk indices involve identifying a set of risk factors (e.g. low maternal education, parental worklessness, teen parenthood, maternal depression), dichotomizing them (as extant or not) and adding them to derive a risk score for each individual in a given sample, combining multiple risks into a single index [3, 5]. Using a cumulative risk index does however not consider how different risk factors combine in individual lives and how they work together in shaping developmental outcomes. It might for example be possible that different combinations of risks are related differentially to distinct outcomes. For example, there is consistent evidence to suggest that early experiences of socio-economic risk are more strongly associated with subsequent educational attainment and subsequent labour market experiences, while the experience of psycho-social family adversity, such as maternal depression or family instability is more strongly associated with emotional and behaviour adjustment [20–22]. Moreover, by relying on mean-based variable centred approaches and collapsing across multiple indicators of adversity, important information might be lost regarding specific challenges faced by different families [10].

### Person-centred approaches

Aiming to gain a better understanding of the inter-relations among risk factors we adopt a person-centred approach which enables us to model constellations of risk. Moving beyond a variable-oriented perspective with a focus on aggregate statistics, a person-oriented analytical approach is useful to capture the configurations of factors that jointly explain behavioural processes and to identify heterogeneous subgroups within a population [23]. Person-centred approaches have been identified as being especially appropriate in the study of families and multidimensional risk contexts [10, 24]. In contrast, variable-centred approaches have been criticised for assuming linear risk effects, not taking into consideration that specific risk factors might not represent a risk for all individuals or families in all conditions [23].

In this study we use latent class analysis (LCA) to examine how different socio-economic risk factors combine within families. LCA enables us to identify subgroups in the population that would go undetected in traditional variable-centred approaches [10, 24]. It is important to note that person-centred approaches are not inconsistent with the assumption of cumulative risk. For example, groups characterised by multiple risk factors and multi-dimensional risk contexts are more at risk for subsequent problematic outcomes. Using a person-centred approach, however, allow us to gain a more specific and nuanced understanding of how risks combine within families and how different combinations of risks are associated with different outcomes.

Previous studies have established heterogeneity across indicators of family adversity, and have identified at least four subgroups with distinctive profiles, highlighting the diversity in constellations of family risks (for a review see [10]). These risk profiles generally include one group characterised by low-risk, one group characterised by high levels of risk across many indicators, different groups characterised by combined socio-economic risks (e.g. low education, unemployment, low income) and the presence or absence of psycho-social risks (e.g. maternal depression), and in some instances also groups characterised by high psycho-social and low socio-economic risk. Most of these studies were conducted in the US focusing on constellations of risk in the parent generation. There is however little understanding if risk constellations are also apparent across cultural contexts–and across generations.

## Intergenerational transmission

Our assumptions regarding the intergenerational transmission of risk constellations are informed by a socio-ecological model of human development [7], differentiating between extra- and intra-familial influences. Regarding the mechanisms that give rise to the intergenerational transmission of risk constellations we take into account processes of social change, cumulative resources within the family, and processes of social selection.

### Social change

Processes of social change assume that societal, or population-level changes which are exogenous to the family make the experiences of new generations different from the experiences of earlier generations [25, 26]. Since the 1970s most Western countries have witnessed the transformation of occupational structures (characterised by a decline of manual occupations and increase of administrative jobs), increasing educational participation, changing family forms, and the growth of alternative family structures. Social change can open up new opportunities as well as obstacles for social mobility and can turn lives around. This can involve structural or forced mobility, i.e. where individuals are forced out (or into) certain occupations or social structures, as well as relative or circulation mobility, which refers to the reproduction of chances for inequality and social mobility [25]. Evidence from the UK suggests, that while increasing numbers of young people are participating in higher education and are increasingly accessing professional and managerial positions, their relative chances of moving into higher occupational categories compared to their more privileged peers remained low [27, 28], although there has been an equalizing trend for women [29]. Moreover, while the last decades have brought enhanced living standards and health conditions, they have also brought more uncertainty, more precarious employment conditions and increasing polarization of those with permanent versus temporary work contracts [30], which in turn is associated with increasing psycho-social stresses [31, 32].

### Cumulative (dis)advantage

Unlike theories of social change, which consider the possibility of new opportunities and potential turning points, theories of cumulative (dis)advantage expect the continuation of (dis)advantage in the second generation [15–17]. This theory focuses on the role of intra-family related processes of status transmission. Children growing up in relative disadvantaged families are at an increased risk to experience similar adversities in the second generation. While a favourable relative position is likely to become a resource that produces further relative gains in the second generation, the lack of resources in the parental generation is assumed to be replicated in the second generation. The underlying assumption represents an instance of the social causation perspective which assumes that social conditions in the family of origin lead to variations in socio-economic and psycho-social outcomes among their offspring [6]. The experience of socio-economic adversity and psycho-social stressors undermines optimal parenting practices, which in turn is linked to children’s adjustment problems in multiple areas [33].

### Social selection

The assumption of cummulative (dis)advantage is mostly focused on structural processes, or the systemic tendency of social reproduction [17], paying less attention to the role of societal change or variations in individual chacteristics. An alternative theoretical model assumes that the characteristics of individuals shape both their socio-economic attainments and psycho-social adjustment. The assumption of social selection effects considers the effect of individual differences in cognitive and behavioural adjustment on subsequent socio-economic attainment and psycho-social wellbeing, highlighting variations in developmental outcomes within subgroups of the population [34, 35]. This assumption leads to the statistical expectation that the association between adversity in G1 and adversity in G2 will be reduced or eliminated when considering the role of individual characteristics.

Previous research evidence suggests that indeed both processes of social causation and social selection are involved in the transmission of family adversity [6, 36, 37]. These two processes have been integrated in the interactionist model of intergenerational transmission of adversity [33], suggesting both direct and indirect associations between adversity experienced in G1 and G2, and mediation effects via individual characteristics. In our modelling approach we thus include indicators of behavioural and cognitive adjustment as potential mediator variables based on evidence of their importance as indicators of developmental health in previous studies [38, 39].

## Current study

We investigate how a broad range of socio-economic and psycho-social risks combine and co-occur within the family of origin (G1) and to what extent constellations of G1 risk exposure predict constellations of risk among their offspring (G2). We use latent class analysis (LCA) to identify constellations of risk that are difficult to detect a priori. Following the assumption of heterogeneity in profiles of adversity, we expect (H1) that we can identify distinct subgroups within the parent generation (G1) and among their offspring (G2) with similar profiles across a number of socio-economic and psycho-social risks.

Regarding the patterns and association between risk profiles across generations, we expect that constellations of risk experiences are different in G2 and G1, given social changes in education and employment opportunities, changing family structures and increasing psycho-social stresses (H2). However, taking into account cumulative risk processes we would expect that a lack of socio-economic and psycho-social resources in G1 is replicated in G2, while an initial favourable position in G1 becomes a resource that produces further gains in G2, leading to increasing inequality (H3). Following the assumption of social selection effects (H4), we expect that at least part of the association between G1 and G2 adversity is mediated by individual characteristics, in particular cognitive ability and indicators of behavioural adjustment.

The study contributes towards a better understanding of how different socio-economic and psycho-social risks combine within families, taking into account heterogeneity in risk exposure. Moreover, we examine the transmission of risk patterns across generations, linking early experiences in the family of origin to own adult outcomes, and examining different mechanisms in the transmission of disadvantage.

## Method

### Sample

The study uses data from the 1970 British Cohort Study (BCS70), a longitudinal study following the lives of over 17,000 individuals born in Great Britain in a week in 1970. Follow-up data collections for BCS70 have taken place when the cohort members (CMs) were aged 5, 10, 16, 26, 30, 34, 38, and most recently at 42 years [40]. Information was collected on educational and occupational development, economic situation, family circumstances, health and wellbeing. Until age 16, information was collected from parents, teachers and CMs themselves, while in later waves the latter became the main respondents. For the measures of risk factors among the parent generation (G1) we used data collected in 1970 and 1975 (birth and age 5). This enables the assessment of early-childhood family circumstances. For the assessment of risk factors among their offspring (G2), i.e., the CMs, we use data collected in 2012 (at age 42) for the same indicators as for G1. In some instances, information from earlier waves of data collection was used, in particular regarding time-invariant indicators that were not available in 2012, i.e., non-UK ethnicity, non-English first language, and teenage parenthood. The study samples comprise 17,588 families (G1) who responded between 1970 and 1975 and 11,226 cohort members (G2) who responded between 1996 and 2012.

### Measures

We assessed a total of fifteen indicators of socio-economic and psycho-social risk exposure across the two generations. For G1, all information (except for maternal depression) were collected at the family level (information from both mother and father, unless there was no father present, which in G1 applied to 7% of the families). For G2 the risk factors were assessed for all CMs. All risks were dichotomized to facilitate comparison. The distribution in percent values and related missing values is presented in Table 1. Percentage differences for each measure are reported in the last column of Table 1. Significance testing showed that for most indicators (except for depression), when taken on their own, the differences across the two populations G1 and G2 were significant.

**Table 1:** Descriptives: Indicators of socio-economic risk by generation (G1, G2) and year of data collection.

	1970–1975		2012		% Difference
Generation	G1	N	G2	N	
**Indicator (% on valid cases)**					
Worklessness	7.31	14146	14.12	9727	+93%
Low social class	21.35	17525	13.73	8269	-36%
Low education	40.59	12727	19.42	9834	-52%
No tenure	43.59	13094	22.99	9393	-47%
Crowding	16.80	12943	7.50	9760	-55%
No partner in household	#1		21.29	9832	
Has children	#2		78.30	9678	
3+ children	28.58	17588	21.35	9678	-25%
Teen parent	27.15	17078	8.23	8168	-69%
Single parent	7.36	17179	17.67	9673	+140%
Non-UK ethnicity	7.66	13003	5.40	11224	-29%
Non-English first language	3.35	13100	3.80	11226	+13%
Depression	18.22#3	12878	18.40	8578	+1%
Illness	12.90	12997	15.04	9745	+16%
Smoking	56.87	17540	53.99	9801	-5%
**N** =	17588		11226		

*Note*: Data are from the 1970 British Cohort Study (BCS70). Sample size varies depending on the year of data collection and across variables, indicating level of missingness.

G1: Parent of the cohort members; G2: Cohort members; Additional indicators were necessary for G2 to clearly identify if they have children and if they are single without children.#1 –For G1 the indicator “no partner in the household” is captured by Single parent; # 2 –G1 comprises only parents, i.e. they all had children; #3 –for G1 we only have an indicator for maternal depression

As all longitudinal studies, BCS70 is affected by non-response and item missingness where a respondent fails to provide all the information requested. We thus have variations in response for individual variables. The varying n in Table 1 indicates the level of missingness for each variable.

#### Socio-economic risk

We included a measure of worklessness, defined by indicators of economic inactivity, comprising those who were looking for work, as well as those who were not looking for work because of health problems, disability, or looking after the family. For G1 worklessness was defined at the family level using information of both parents in 1970 and father being unemployed for longer than four weeks in 1975. Low social class differentiates between those in partly-skilled and unskilled occupations (categories IV and V of the Registrar General’s measure of social class, RGSC) [41], versus others (categories IIIM—Skilled manual -, IIINM—Skilled non-manual -, II—Managerial and Technical -, and I–Professional—of the RGSC). Where the father was absent in G1, the social class (RGSC) of the mother was used. Low levels of education in G1 and G2 includes those with National Vocational Qualification (NVQ) at Level 1 or below (equivalent to the international schooling ISCED levels 0 to 2). For G1 we used the highest level of qualification of either parent. Housing tenure in G1 and G2 comprises the proportion of those who rented versus those who were living in their own home (either as homeowners outright or mortgagees). Crowding in G1 and G2 was measured by the ratio of more than one person per room. Teen parenthood for G1 was recorded for those who became a parent before age 20. For G2 we had to compute this time-invariant indicator using the 1996 data (collected at age 26) using a question asking the age of the first child. Large families were coded as 1 when the number of dependent children in the household was equal or higher than three. Single parenthood was coded (1) if child caregivers in G1 and G2 were not living with a partner. In G2 two additional indicators had to be used to identify those who have children (has children) and to differentiate single parents from those who live alone with no children (presence of a partner in the household versus no partner in household). Non-British ethnicity was identified as belonging to ethnic groups different from White British, including Irish (only 47 cases in G2). We also recorded language generally used at home (Non-English as the first language spoken at home).

#### Psycho-social risks

*Depressive symptoms* were measured with the Rutter Malaise inventory (RMI) [42] which in 1970 was assessed of mothers of the cohort members only. In G1 the full 24 yes-no items were used, with a score ranging from 0 (no symptoms) to 23 (highest level of depression). Individuals responding ‘yes’ to eight or more of the 24 items are considered to be at risk of depression [43]. In 2012 only nine of the 24 questions were asked of G2. The nine-item modified RMI scale has shown to correlate well with both reported depression and anxiety [44]. In this shortened version individuals responding yes to four or more of the 9 items are considered to be at risk of depression. *Illness* in G1 was assessed in 1975 and in G2 in 2012, asking whether there has been any case of severe or prolonged illness, handicap or disability. *Smoking* habits in G1 were measured by parental report of being a smoker in 1970 and 1975 (defined as smoking an average of one or more cigarettes a day). For G2, in 2012 the CMs were asked whether they had ever been smokers.

*Behavioural adjustment* was measured in 1975, when the CMs were aged 5, using a modified version of the Rutter A-Scale tapping into the three dimensions of emotional adjustment, conduct, and attention problems [42]. The Rutter A-Scale has good test–retest reliability [45]. Emotional problems are assessed by items such as: the child is often worried/worries about many things, and often appears miserable, unhappy, tearful or distressed. Examples for items to assess conduct problem are: the child is often disobedient, often destroys own or others’ belongings, and frequently fights or is extremely quarrelsome with other children; and for attention/hyperactivity problems: the child is squirmy, fidgety child, cannot settle to anything for more than a few moments. Items are assessed on a scale from 0 to 2 (does not apply, applies somewhat, certainly applies). A factor analysis confirmed the existence of the three main factors, with satisfactory Alpha coefficients of 0.83 (conduct), 0.72 (emotional), and 0.82 (hyperactivity) for the different subscales. The scale scores were z-standardized with a high score indicating behavior problems.

*Cognitive ability* was assessed in 1975 at age 5 through the Copying Designs Test: An assessment of visual-motor co-ordination [42]; the English Picture Vocabulary Test: A test of verbal vocabulary [46], and the Human Figure Drawing (Draw-a-Man) Test: reflecting conceptual maturity [47, 48]. All these tests have high reliability and validity, and correlate well (r = >0.5) with standard IQ tests such as the Wechsler and Binet test [49]. The test scores from the three tests were summarized by a single standardised, uni-dimensional factor score of childhood general cognitive ability.

*Control variables*. In the multivariate analysis we adjust for gender of the CMs in order to account for gender-based differences in experiences of (dis)advantage and associated processes.

#### Analytic strategy

We used LCA to identify subgroups of families with similar risk profiles. LCA is a data-reduction technique similar to factor analysis [50] appropriate for the assessment of population heterogeneity in multivariate fashion [51] and can be used to identify typologies of unobservable, i.e. latent, classes of risk. We performed two separate LCA analyses, one for each generation. The LCA analyses were conducted in Mplus [52]. To identify the optimal number of latent classes, we examined the fit statistics of different models specifying incrementally larger number of classes. Measures of model fit include the log-likelihood value (LL), the Akaike Information Criterion (AIC), the sample-adjusted Bayesian Information Criterion (s-BIC). For the first index (the LL), the higher the value the better the solution, whilst the opposite is true for the AIC and s-BIC. An additional indicator of model fit is the entropy measure, assessing the quality of the classification: values above .800 are generally desirable [53]. Finally, search for the optimal solution is guided by the p-value of the adjusted Lo-Mendel-Rubin likelihood ratio test (Adjusted LRT), which compares the appropriateness of the last estimated model with *k* classes with the previous one with *k*-1 classes [54, 55], as well as consideration of the parsimony and meaningfulness of the solution.

To account for missingness in the data we performed LCA in Mplus v 8, and generated multiple imputed data using the estimation-maximisation (EM) algorithm, which optimizes the complete-data maximum-likelihood (ML) estimator via an accelerated iterative estimation procedure in a full-information maximum likelihood fashion (FIML) [52]. To exploit the maximum amount of information available in the data we used all the available cases for G1 in waves 1970 and 1975 (n = 17,588), and for G2 we used all cases available from waves conducted between 1996 to 2012 (n = 11,226). An analysis of response bias showed that the achieved samples at age 42 did not differ from the target sample across a number of critical variables (social class, parental education, and gender), despite a slight under-representation of males, especially those whose parents were single in 1970 [56].

In a next step, we used transition matrix analysis [57] to assess the unconditional intergenerational transmission of risk constellations from G1 to G2, where similarities in constellations of risk between G1 and G2 are described. We then ran a multinomial regression model [58] using G2 latent classes as the outcome variable, based on the 11,226 cases available. To that aim, the Mplus LCA grouping results were imported into STATA15, which gives the option of augmented regression models for categorical variables [59]. In particular, it provides marginal effects which give a better understanding of the influence and the size of effect for each predictor variable. To account for both item- and unit-missingness in the predictor variables used in the regression model, we used multiple imputation [60] by chained equations as implemented in STATA15.

## Results

### Constellations of risk in G1

Deciding on the number of classes we were guided by the model fit indices, the parsimony of the model as well as the interpretability of the identified groups. For G1 the entropy index (Table 2) suggests the 4-class solution to be optimal–while the Adjusted LRT suggests no significant improvement beyond a 9-class solution. Inspection of the Log-likelihood statistics, the AIC, and s-BIC suggests a significant increase of model fit for the 3-class and 4-class solutions. On balance, we opted for the 4-class model for G1, confirmed by the inspection of solutions with more than four classes, which did not reveal any qualitatively different configurations, or were not interpretable. For example, the 5-class solution divided a low risk group into slightly different combinations of low risk.

**Table 2:** Selection of number of classes for the parents’ generation (G1). Indicators of Model Fit.

Number of classes	Log-Likelihood	Model’s free parameters	AIC	S-BIC	Entropy	*A*-LRT p-value for K-1 classes
2	-82159.706	27	164373.413	164497.532	0.563	0.0000
3	-81201.983	41	162485.967	162674.445	0.705	0.0000
4	-80960.157	55	162030.314	162283.151	0.761	0.0000
5	-80798.753	69	161735.507	162052.702	0.626	0.0000
6	-80690.859	83	161547.719	161929.272	0.655	0.0057
7	-80620.328	97	161434.655	161880.568	0.611	0.0024
8	-80562.343	111	161346.687	161856.957	0.632	0.0000
9	-80540.182	125	161330.363	161904.992	0.569	0.068

*Note*: AIC = the Akaike Information Criterion; S-BIC = sample-adjusted Bayesian Information Criterion; A-LRT = adjusted Lo-Mendel-Rubin likelihood ratio test.

The interpretation of the four selected latent classes is aided by the inspection of Table 3, showing the conditional response probabilities (ranging from 0 to 1) for each latent class, which indicate to what extent each class is defined by the different risk factors. A probability of 0 means that the likelihood of belonging to that specific latent class is zero for people for whom the specific characteristic applies, whilst 1 represents the highest likelihood.

**Table 3:** Conditional response probabilities by latent class for G1.

	Class: Response probabilities			
Class	High-risk Large Families	High-risk Single Parent	BEM	Low-risk
Worklessness	**0.197**	**0.123**	**0.053**	**0.019**
Low class	**0.363**	**0.395**	**0.411**	**0.075**
Low education	**0.753**	**0.653**	**0.626**	**0.176**
No tenure	**0.827**	**0.785**	**0.198**	**0.168**
Overcrowding	**0.121**	**0.025**	**0.183**	**0.001**
3+ Children	**1**	**0**	**0.579**	**0.191**
Teen parent	**0.495**	**0.472**	**0.323**	**0.110**
Single parent	**0.088**	**0.201**	0.009	**0.014**
Non-UK parent	**0.066**	**0.054**	**1**	**0.041**
Non-English first language	**0.008**	**0.005**	**0.824**	**0.012**
Depression	**0.320**	**0.274**	**0.280**	**0.092**
Illness	**0.162**	**0.140**	**0.136**	**0.113**
Smoker	**0.785**	**0.722**	**0.297**	**0.448**
**Class Probabilities in %**	*16*.*3*	*24*.*0*	*2*.*1*	*57*.*6*

*Note*: Probabilities in bold are statistically significant. BEM = British ethnic minority. Significance is established at the 95% confidence level.

The majority of parents in G1 are clustered into a class, which we labelled as ‘low-risk’ (57.6%), showing a relative low probability of socio-economic disadvantage, and a low risk for physical and psychological health risks (except for smoking) compared to the other groups. A second class, identified as ‘high-risk single parents’ comprised 24% of the population. This class is characterised by high probabilities of socio-economic risk in terms of low education, no housing tenure, low occupational class, teen and single parent status as well as a relative high health risks compared to the low risk group. Interestingly, this group is least likely to have 3+ children. A third latent class was labelled as ‘high-risk large families’ comprising 16.3% of the population. This class is characterised by the highest likelihood of having three and more children, being workless (compared to the other groups), with a high probability of low education, living in rented accommodation, being a teen parent, suffering from depression, illnesses and being a smoker. A fourth class comprised British ethnic minority families (BEM) and was labelled as ‘BEM parents’ (2.1% of the population) characterised by relative low levels of education, large family size, low social class, living in overcrowded conditions, and not using English as their first language. This class also had a relative high-risk of depression as compared to the low-risk class, but had the lowest probability of being smokers as compared to all the classes. Moreover, parents belonging to this class are more likely to be in work and own their own home than those in the high-risk and single-parent classes.

### Constellations of risk in G2

Based on the model fit statistics for different k-classes solutions (Table 4), as well as at their interpretability, we opted for the 5-class grouping for G2. Response probabilities for each indicator of socio-economic and health risk factors for G2 are shown in Table 5.

**Table 4:** Selection of number of classes for the CMs’ generation (G2). Indicators of Model Fit.

Number of classes	Log-Likelihood	AIC	Sample-adjusted BIC	Entropy	Adjusted LRT p-value for K-1 classes
2	-58779.237	117620.473	117754.350	0.594	0.000
3	-57274.030	114642.061	114845.035	0.662	0.000
4	-56729.225	113584.450	113856.521	0.649	0.000
5	-56236.274	112630.547	112971.716	0.654	0.000
6	-55834.960	111859.919	112270.185	0.686	0.000
7	-55628.341	111478.682	111958.046	0.684	0.053

*Note*: AIC = the Akaike Information Criterion; S-BIC = sample-adjusted Bayesian Information Criterion; E = Entropy; A-LRT = adjusted Lo-Mendel-Rubin likelihood ratio test.

**Table 5:** Conditional response probabilities by latent class for G2.

	Single parent	Large family	High SE and health risk	Low-risk	Low-risk no children
Worklessness	**0.123**	**0.268**	**0.934**	**0.073**	**0.053**
Low class	**0.225**	**0.277**	**0.351**	**0.100**	**0.114**
Low education	**0.251**	**0.372**	**0.574**	**0.119**	**0.151**
No tenure	**0.475**	**0.446**	**0.830**	**0.067**	**0.215**
Overcrowding	**0.060**	**0.450**	**0.107**	**0.028**	**0.008**
No partner	**0.670**	***0***	**0.591**	***0***	**0.416**
Has children	***1***	***1***	**0.737**	***1***	**0.066**
3+ Children	**0.242**	**0.724**	**0.299**	**0.186**	***0***
Teen parent	**0.195**	**0.290**	**0.240**	**0.040**	***0***
Single parent	**0.780**	**0.173**	**0.285**	**0.081**	***0***
Non-UK ethnicity	**0.055**	**0.219**	**0.032**	**0.026**	**0.049**
Non-English first language	**0.023**	**0.188**	0.012	**0.02**	**0.026**
Depression	**0.180**	**0.226**	**0.703**	**0.131**	**0.171**
Illness	**0.118**	**0.171**	**0.871**	**0.086**	**0.136**
Smoker	**0.690**	**0.650**	**0.802**	**0.459**	**0.515**
**Class Probabilities in %**	10.1	8.9	4.0	62.0	15.1

*Note*: Probabilities in bold are not statistically significant. Significance is established at the 95% confidence level. SE = socio-economic.

The largest class ‘low-risk families’ comprises 62% of the sample, and is characterised by low levels of socio-economic and psycho-social risk, as well as by having children. Another low-risk class comprises mostly cohort members without children (15.1% of the G2 sample), and was labelled ‘low-risk no children’. This group is also characterised by relative low levels of socio-economic risks, an increased probability of being single, and largely includes CMs without children. A third class (comprising 10.1% of the G2 sample) is characterised mostly by an increased probability of being a ‘single parent’, with moderately high likelihood of being in low education and occupational status, with no housing tenure, no partner, and being a smoker. A fourth class comprising 8.9% of the sample includes CMs with more than three children (‘large families’), relative low level of education, more likely to be renting than the low-risk families, to live in overcrowded accommodation, to be a teen parent, not from a white-British background and with a language different from English as the main one spoken at home. A fifth class consists of CMs characterised as experiencing ‘high levels of socio-economic and psycho-social risk’, comprising 4% of the sample. This group shows the highest levels of worklessness, low levels of educational and occupational status, lone parenthood, no housing tenure, overcrowding, as well as a high risk of depression and ill health.

### Intergenerational transmission of risk constellations

Table 6 gives the transition probability matrix describing the degree of intergenerational mobility. The findings suggest considerable levels of social mobility as the majority of G2 are able to avoid the experience of high levels of risk. For instance, 54% of G2 from a high-risk family background become a low-risk family themselves and 11% are found in the low-risk no children latent group. However, the proportion of G2 from low-risk families becoming low-risk families themselves is considerably larger than for G2 cohort members from high-risk large families, high-risk single parent families or BME parents.

**Table 6:** Transition probability matrix G1 → G2 latent classes.

		G2 Latent class					
		Single parent	Large family	High socio-economic and health risk	Low-risk	Low-risk no children	Total
G1 Latent class	High-Risk Large Families	13.41	10.82	5.39	57.55	12.83	100
	High-Risk Single Parent	8.17	29.18	4.67	46.3	11.67	100
	BEM parents	8.09	5.20	2.35	67.13	17.23	100
	Low-risk	10.14	8.28	3.99	62.46	15.13	100
	Total						

*Note*: BEM = British ethnic minority

The associations between group membership across generations were further tested by means of stepwise multinomial regression models, using the G1 ‘low-risk’ class as the reference group. Table 7 shows the average marginal effects as predicted probabilities of G2 groups’ membership, with significant coefficients marked in bold. Marginal effects provide a good approximation to the amount of change in predicted probabilities due to a change in a particular predictor, taking into account all predictors included in the model. Model 1 (not including the individual characteristics) shows an increased probability of being in the G2 ‘high-risk’ group for cohort members from G1 ‘high-risk families’ (by 5.5 percentage points) and G1 ‘high-risk single parent’ groups (by 3 percentage points) compared to the reference group, i.e. those from the G1 ‘low-risk’ group. Likewise there is an increased probability for G2 from G1 high-risk families and G1 single parent families to be in the G2 single parent group. The probability of entering the G2 ‘large family’ group is increased for those from G1 ‘high-risk families’, ‘high-risk single parent’ and ‘ethnic minority’ groups. The probability of entering the G2 ‘low-risk family’ and ‘low-risk no children’ group is significantly lower for cohort members from the G1 ‘high-risk families’, high-risk single parent families’ and ‘BME parents’ than for those from the ‘low-risk’ group, suggesting a considerable degree of continuity of advantage.

**Table 7:** Marginal predicted probabilities of G2 latent class membership by predictors.

Outcome: G2 Latent class	G2 Single parent	G2 Large family	G2 High-risk	G2 Low-risk	G2 Low-risk single parent
**G1 Predictors**	**Model 1**				
	Predicted Prob	Predicted Prob	Predicted Prob	Predicted Prob	Predicted Prob
**G1 Latent classes** **(Ref: Low-risk)**					
**High-risk**	**0.052**	**0.086**	**0.055**	**-0.131**	**-0.062**
**St. Err.**	0.008	0.008	0.006	0.012	0.008
**Single parent**	**0.053**	**0.060**	**0.030**	**-0.098**	**-0.045**
**St. Err.**	0.007	0.007	0.005	0.011	0.008
**British ethnic minority**	-0.001	**0.249**	0.022	**-0.214**	**-0.056**
**St. Err.**	0.017	0.028	**0.013**	0.031	0.020
	**Model 2**				
**G1 Latent classes** **(Ref: Low-risk)**					
**High-risk**	**0.041**	**0.073**	**0.040**	**-0.101**	**-0.053**
**St. Err.**	0.008	0.008	0.006	0.013	0.009
**Single parent**	**0.043**	**0.050**	**0.021**	**-0.077**	**-0.038**
**St. Err.**	0.007	0.007	0.004	0.011	0.008
**British ethnic minority**	-0.008	**0.216**	0.008	**-0.175**	-0.041
**St. Err.**	0.017	0.028	0.010	0.032	0.023
**G2 Gender Female** **(Ref: Male)**	**0.078**	**0.016**	**0.010**	**-0.067**	**-0.037**
**St. Err.**	0.006	0.005	0.003	0.009	0.006
**G2 Rutter’s Emotional adjustment problems age 5**	**-0.008**	-0.004	0.000	0.003	**0.009**
**St. Err.**	0.003	0.003	0.002	0.005	0.004
**G2 Rutter’s Conduct problems age 5**	**0.009**	0.004	**0.005**	-0.005	**-0.012**
**St. Err.**	0.003	0.003	0.002	0.005	0.004
**G2 Rutter’s Attention problems age 5**	0.006	0.005	**0.008**	**-0.018**	-0.002
**St. Err.**	0.003	0.003	0.002	0.005	0.004
**G2 Cognitive ability age 5**	**-0.009**	**-0.011**	**-0.011**	**0.025**	0.006
**St. Err.**	0.002	0.002	0.001	0.004	0.003

*Note*: Average marginal effects as Predicted probabilities (Prob) in bold are significant at the 95% confidence level. St. Err. = standard error; Reference category for G1 latent classes is low-risk; Reference category for G2 latent classes is low-risk. G1 = generation 1; G2 = generation 2.

Adding the individual characteristics reduces the coefficients considerably. For cohort members from G1 British ethnic minority groups the coefficient regarding entry to the G2 low-risk single parent group even becomes non-significant. Females are generally less likely to be in the groups involving low levels of risk, and have a higher probability to be in groups characterised by moderate to high socio-economic and psycho-social risk (model 2). Early emotional adjustment problems are associated with a lower risk of being in the G2 single parent group and a higher likelihood of being in the G2 low-risk no children group (maybe reflecting hesitations in making the step into family formation). High levels of conduct problems are associated with an increased risk of being in the single parent or the high-risk group and a reduced likelihood of being in the low-risk singles group, while high levels of attention problems are associated with increased probability of being in the high-risk group and reduced likelihood of being in the low-risk groups. High cognitive ability is associated with increased probability of being in the low-risk family group and reduced probability of being in a high or moderate risk groups.

## Discussion

This study examines the intergenerational transmission of disadvantage. The findings demonstrate the usefulness of a person-centred approach for modelling the interlinkages and combination of multiple risk factors within the family context. We could identify different distinct constellations of socio-economic and psycho-social risks in both the parent (G1) generation and their offspring (G2), confirming assumptions of heterogeneity in risk profiles (H1). Moreover, the findings confirm the usefulness of a socio-ecological approach, emphasising significant influences of both intra- and extra-familial factors, as well as the role of individual characteristics in the intergenerational transmission of risk.

Using latent class analysis (LCA) in a large, nationally representative sample, we could identify a number of distinct risk profiles that capture parsimoniously the variability and the intersection of the multiple risk factors in both the family of origin (G1) and family of destination (G2). The findings support previous studies establishing at least four to five subgroups with distinctive profiles [10].

Regarding the processes of intergenerational transmission of risk profiles we find evidence of processes of social change, of cumulative (dis)advantage and social selection. Support for processes of social change (H2) is evident in that the constellations of risks differed across the generations and in the emergence of new risk patterns. For example, the G1 group dominated by ethnic minority families is not repeated in G2, suggesting processes of social integration. In addition we find a decrease in high-risk groups, and an increase in moderate and low-risk groups, which in the second generation also include a ‘low-risk no children’ group (15%) who by age 42 have not yet committed themselves to family formation. We have to await future waves of data collection to see if this group reflects a postponement of commitment or if they forgo it completely.

Confirming previous studies [24] we find considerable levels of social mobility. However, the findings suggest structural rather than relative mobility. Despite a considerable proportion of G2 growing up in relative disadvantaged families avoiding the experience of high level risks when they are adults themselves, the probability for G2 cohort members born into high-risk families to also encounter high levels of both socio-economic and psycho-social risk in their own lives is significantly raised and the probability for entering any of the two low-risk groups is reduced compared to cohort members growing up in low-risk families. The findings thus points to processes of cumulative advantage (H3) and persistent social inequalities. The findings also suggest an increasing polarisation, i.e. a trend towards higher levels of education and new employment opportunities among many and at the same time increased levels of socio-economic and psycho-social risks among the most disadvantaged of the G2 sample (4% in this study). The emergence of such pockets of deep risk is potentially associated with permanent exclusion or peripheralization–an insider-outsider phenomenon identified in labour market economics [30].

In addition we find evidence for social selection effects (H4), suggesting that individual characteristics such as behavioural adjustment and cognitive ability at least partly mediate the association between G1 and G2 constellations of risk. Future studies have to explore these interactions in more detail, identifying distinct risk and protective factors and processes potentially enabling individuals to escape the vicious cycle of intergenerational disadvantage.

The contribution of this study is to highlight the joint effect of both social causation and social selection processes, as emphasized in the interactive model of intergenerational family disadvantage [33]. Moreover, our findings suggest that the interactive model should be extended to a socio-ecological model including a focus on processes of social change, taking into account that in addition to individual factors both family specific and external influences from the wider social context influence one’s life course [6,37].

In interpreting the findings we have to be aware not to reify the meaning attached to a latent class or the label assigned to it [51]. The final models provide only a summary of the many ways in which constellations of risk may occur in society. In selecting the number of classes we chose the most parsimonious model, comprising four classes in G1 and five in G2, which provided a useful taxonomy of risk patterns. Adding more classes might have improved the BIC, yet there was little relative gain in overall model fit.

The risk configurations of G1 and G2 only represent a snap shot in time, and do not capture the dynamics of family transitions. For example, ‘moderate risk single parents’ could become ‘low-risk families’ or the ‘high-risk’ at a subsequent observation point. Future work should build on advances in latent transition research [51] to examine changes in risk constellations over time in more detail. In our analysis we assess risk exposure in G1 when most parents were aged around 30 years, while their offspring was aged 42. We compared constellations of risk in G2 during their 30s, but found that G2 by age 42 was more similar to G1 (especially regarding family formation).

We only captured a selection of risks and not all possible risk facing families today. For example, due to limitations in the data we had no measure of family income for G1. Nor could we include indicators of parental health, and future research has to examine in more detail the role of paternal health in defining risk clusters and in shaping subsequent outcomes. Moreover, the inclusion of different sets of risk and the use of different dichotomization or risk factors measured as continuous indicators may shape the number and types of groups that emerge. The findings are thus specific to the sample and the risk factors included in the model. However, they should be interpreted within the context of other recent studies using LCA which identified similar constellations of family risk within the US context [10]. Together, the findings contribute towards a better understanding of how risks co-occur and are transmitted across generations. The relationships established in this study are not causal and there are other potential family factors and processes related to risk profiles in G1 and G2 and how they are linked over time.

## Conclusion

Despite its limitations, this study makes several contributions. First, we move beyond traditional variable-based models and cumulative risk models by applying a person-centred approach to identify constellations of both socio-economic and psycho-social risks and examine how they are transmitted across generations. To our knowledge this is the first study to examine constellations of risks in two generations, providing a better understanding of a) how risks combine in families; b) emerging risk constellations in times of social change; c) the extent to which constellations of risk are transmitted to the second generation; and d) the joint influence of processes of cumulative advantage, social selection and social change. The findings suggest that meaningful risk constellations can be identified. We find support for the assumption of processes of social change that can open up new opportunities, especially regarding education and employment opportunities, but also new risks, and potentially an increasing peripheralization of the most vulnerable families. There is evidence of social mobility–although structural rather than relative mobility—indicating processes of cumulative advantage. In addition, the findings point to the role of individual characteristics and processes of social selection in shaping the intergenerational transmission of family disadvantage. Adopting a socio-ecological model provides a more comprehensive understanding of the multiple influences involved in the transmission of risk constellations from one generation to the next, considering the influence of individual characteristics, family resources and external influences from the wider social context in shaping one’s life course. The findings reported here are a starting point for a better understanding of variations in risk exposure across families and across generations, and how risks are transmitted from one generation to the next.
